# Central and peripheral nervous system involvement caused by Zika and chikungunya coinfection

**DOI:** 10.1371/journal.pntd.0005583

**Published:** 2017-07-13

**Authors:** Carlos A. A. Brito, Fernanda Azevedo, Marli T. Cordeiro, Ernesto T. A. Marques, Rafael F. O. Franca

**Affiliations:** 1 Department of Clinical Medicine, Federal University of Pernambuco - UFPE, Recife, Brazil; 2 Department of Neurology, Santa Joana Hospital, Recife, Brazil; 3 Department of Virology and Experimental Therapy, Oswaldo Cruz Foundation - FIOCRUZ, Aggeu Magalhães Institute, Recife, Brazil; 4 Department of Infectious Diseases and Microbiology, University of Pittsburgh, Center for Vaccine Research, Pittsburgh, Pennsylvania, United States of America; Colorado State University, UNITED STATES

## Background

Large outbreaks of dengue, Zika, and chikungunya are taking place in several countries in Asia and Latin America [1,2], and simultaneous circulation of these 3 arboviruses in the same region raises the possibility of coinfections of vertebrate and invertebrate hosts. Indeed, several cases of dengue and chikungunya coinfections have been reported [3]. However, cocirculation of Zika and chikungunya is more recent. The 2013 outbreak in the French Polynesia [4] and the 2015 outbreak in Brazil [5] are the largest Zika virus outbreaks described to date. In Brazil, the Zika and chikungunya outbreaks partially overlapped, and few reports of coinfection are present in the literature [6,7]. Here, we report a severe case of meningoencephalitis associated with peripheral polyneuropathy in a 74-year-old patient due to a coinfection with Zika and chikungunya.

## Case report

On April 3, 2016, a 74-year-old male resident from Recife, northeast Brazil, with history of systemic arterial hypertension and controlled diabetes mellitus type II was admitted in a private hospital in the city of Recife, Pernambuco, in northeast Brazil. The patient appeared with acute symptoms of fever; arthralgia in his hands, knees, and ankles; and edema in the feet. The patient was admitted for hydration and prescription of symptomatic medications. On the third day from the onset of the symptoms, the patient began to vomit and worsen in overall health status.

During hospitalization, disorientation and agitation were also added to the clinical presentation, followed by refractory arterial hypotension and shock, despite adequate volume reposition. The patient developed hypoxia, which required mechanical ventilation and use of vasoactive drugs. Complete blood count (CBC) showed normal leukocytes, 8 x 10^9^/L, with 88% of polymorphonuclear leukocytes and platelet count of 90 x 10^9^/L. Initial neurologic evaluation was performed at the intensive care unit with the patient sedated. However, the presence of stiff neck and muscular weaknesses associated with global areflexia were clear in the neurological examination. Altogether, these findings suggested the diagnosis of acute meningoencephalic syndrome. Laboratory analyses of cerebrospinal fluid (CSF) demonstrated the presence of a total 85 cells—60% lymphocytes, 20% monocytes, 20% neutrophils; protein 251 mg/dL; glucose 133 mg/dL; negative VDRL. Laboratory-performed ELISA IgM and PCR for routinely tested pathogens (cytomegalovirus (CMV), dengue virus, HIV-1, human T-lymphotropic virus (HTLV), schistosomiasis, cysticercosis, herpes simplex virus type 1 and 2) were negative.

**Fig 1:**
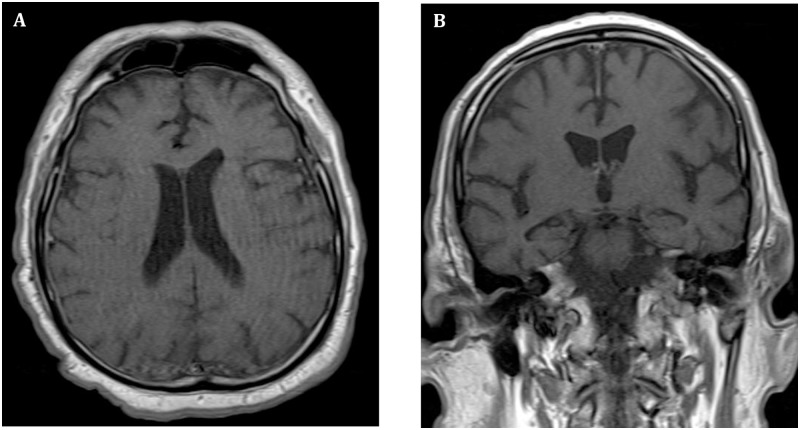
Brain MRI from a 74-year-old patient with reverse transcription–polymerase chain reaction (RT-PCR)–confirmed Zika and chikungunya coinfection. **(A)** T_1_–weighted images of the head were obtained after the administration of contrast material. Axial and coronal T1 spin-echo contrast-enhanced MRI showing diffuse dural bilateral meningeal enhancement. **(B)** Absence of leptomeningeal enhancement.

Cranial magnetic resonance imaging (MRI) showed a diffuse bilateral dural meningeal enhancement with no evidence of leptomeningeal enhancement (Fig 1A and 1B). Following sagittal T1 fat-suppressed spin-echo contrast-enhanced MRI, leptomeningeal enhancement at the anterior region of the bulb-medullary transition was detected (data not shown). Medical support treatment associated with antiviral and intravenous administration of acyclovir was initiated while the etiological investigation continued. After systemic clinical improvement, nervous system depressant drugs were suspended and a new neurological evaluation was performed. The neurological examination confirmed the existence of asymmetrical severe muscle weakness on both upper and lower limbs, mainly in the distal segment, accompanied by absent deep tendon reflexes, paresthesia in the hands and feet, pain in the back and extremities, and mild sensory abnormalities.

Electromyography revealed demyelinating polyradiculoneuropathy (sensitive and motor) with axonal damage. The exam also indicated signs of severe conduction block in distal segments, most evident on the left side. The electromyography, in addition to the clinical symptoms, was consistent with a diagnosis of acute inflammatory demyelinating polyneuropathy (AIDP), the most prevalent form of Guillain-Barré syndrome (GBS). CSF was collected, and an investigation of dengue, Zika, and chikungunya viral RNA was performed by Real Time quantitative RT-PCR (RT-qPCR) employing virus sequence-specific probes. The results evidenced the presence of Zika and chikungunya viral RNA on CSF. An IgM ELISA test specific for chikungunya virus (EuroImmun, Lübeck, Germany) was also positive in the CSF; however, the results of IgM ELISA for Zika and dengue viruses were both negative on CSF. Additionally, dengue immunoglobulin G (IgG) was positive on serum; however, dengue and Zika IgM were negative on serum. Based on that, definitive laboratory findings supported the diagnosis of an acute chikungunya and Zika virus simultaneous coinfection.

The patient was treated with intravenous human immunoglobulin (IVIG) 0.2g/kg/day for 5 days and evolved with progressive neurological improvement and withdrawal of mechanical ventilation. At 6 months after admission, the patient presents a significant clinical improvement. However, strength deficiency in lower limbs persists, especially on the left side. He now presents urinary retention and needs to use an intermittent catheter for relief. Sensory abnormalities and pain symptoms are no longer a problem, and he’s functionally independent.

## Discussion

Chikungunya was initially described in 1952 in Tanzania. However, in 2005, the virus reemerged in great outbreaks in the Indian Ocean islands, especially on Reunion Island, where one third of the population was infected [1,8]. The first important Zika virus outbreak was documented in the Yap Islands in 2007 [9] and a larger outbreak was reported in 2013 in the French Polynesia [4]. In Brazil, the first chikungunya epidemic documented wave occurred in July 2014. Subsequently, Zika virus emerged with a massive outbreak in the first semester of 2015 [10–13] and a second, much larger wave of chikungunya occurred in 2016. In spite of chikungunya and Zika outbreaks, dengue virus is still the most important arboviral disease in the world. Thus, considering the circulation of all 4 serotypes of dengue virus in Brazil, large dengue outbreaks were common over the last decades. Based on that, the triple epidemic has become a reality, with simultaneous circulation of these 3 viruses [14] (or 7, if you count the 4 different dengue serotypes separately). In this report, we present a severe case of a disease with simultaneous involvement of central and peripheral nervous systems and a discussion about the potential risk of arboviral coinfections to amplify the severity of the disease and to generate atypical clinical presentations.

The clinical presentation of this chikungunya and Zika coinfection case confirmed simultaneous involvement of central and peripheral nervous systems. Although neurologic disturbances associated with several arboviruses were recognized, there is a much higher frequency in neurological impairment following chikungunya and Zika infection than those resulting from dengue virus infection. In fact, chikungunya and Zika can lead to different types of neurological involvement. While the reports associated with chikungunya have a prevalence of central nervous system (CNS) involvement (especially meningitis and encephalitis) [15], Zika has a higher frequency of peripheral disease, especially GBS [16]. Thus, a concomitant peripheral and CNS disorder becomes a rare opportunity for the study of uncommon arboviral disease manifestations.

Currently, there are only a few reports describing neuroimaging findings of neurological injury associated with chikungunya. Chusri et al. 2011 [17] reported neurological abnormalities in patients with chikungunya infection. In 1 patient, the main brain MRI abnormality was located in the subcortical white matter, with the presence of multiple tiny hyperintense dots in T2-weighted and fluid-attenuated inversion recovery images. On another patient, MRI and electromyography confirmed acute progressive quadriparesis and myeloneuropathy, indicating an acute sensorimotor peripheral neuropathy typical for demyelinating disease. In the case presented in this report, we confirmed chikungunya and Zika infection based on virus RNA detection by RT-qPCR in CSF and chikungunya IgM detection also in the CSF. Acute meningoencephalitis syndrome was diagnosed based on CSF cellular and protein contents, demonstrating the presence of inflammatory cells. In fact, Zika and chikungunya viruses are known to be highly neurotropic, and direct viral replication on the CNS contributes to the neurologic manifestations that are seen during infection. Thus, an inflammatory profile is expected following viral replication on the CNS.

The reports of chikungunya and Zika coinfection are very limited [6,18,19]; this can be explained by the fact that only recently has Zika virus been causing large outbreaks. Thus, the clinical manifestations of Zika and chikungunya coinfections require further investigation. Recently, a case of a pregnant woman with simultaneous detection of dengue, Zika, and chikungunya without any repercussion to the fetus was published. The mother presented the following clinical symptoms: nonpurulent bilateral conjunctivitis; intense upper limbs, thorax, and abdomen pruritic maculopapular rash; headache; mild-to-intense bilateral metacarpophalangeal and wrist arthralgia; and limb edema. Interestingly, no neurological complications were reported in this patient [6]. Moreover, Waggoner et al. reported the identification of 16 cases (4.6%) of Zika and chikungunya coinfections in a group of 263 patients presenting arbovirus symptoms. According to the authors, clinically, the patients with coinfections and monoinfections presented a very similar pattern; the only difference was a slightly higher hospitalization rate in patients with coinfections (83.3%) versus monoinfections (64.0%) [19]. Only recently, in a publication in July 2016, 3 cases of coinfection with neurological complications were notified in Ecuador. Among these 3 coinfections cases, 1 developed GBS [7]. Real Time RT-qPCR laboratory analysis confirmed this case as being positive for Zika and chikungunya based on viral RNA detection in serum and CSF. Additionally to these reports, 7 fatal cases with dengue and chikungunya virus coinfection were reported in Colombia from 2014 to 2015. Although these cases were characterized by variable clinical and laboratory characteristics, only hemorrhagic and no neurological manifestations were documented on these patients [18]. To our knowledge, among neurological complications following chikungunya infection, encephalitis is the most common reported clinical characteristic [15]. Thus, although chikungunya and dengue coinfection cases have been described, most of the studies comparing the clinical significance of coinfections have not demonstrated any type of disease exacerbation or any differential clinical outcome (relative to single infections) [20–22]. Despite the fact that the patient from the case presented here is 74 years old and survived an acute coinfection, previous reports have shown that age could be considered a risk factor for neurologic complications and mortality for chikungunya infection [23,24].

In conclusion, the case presented here points out the need to monitor arboviruses coinfection, especially for 2 important viruses with well-known neurotropism, such as Zika and chikungunya. Since these viruses are now endemic in several countries, we should be aware of more neuro-coinfection cases in the future. Although data on literature is still scarce, we cannot exclude the possibility that coinfections could lead to more severe neurological damage. In fact, the case here presented reinforces the need of arboviruses effective surveillance programs in recently affected areas, especially in those that are now epidemic for Zika. Also, our data alert for a potential risk of more severe diseases as a result of simultaneous infections.

## Ethics statement

Ethics approval for the study was obtained from the Oswaldo Cruz Foundation—FIOCRUZ, Aggeu Magalhães Institute, Ethics Committee under the numbers CAE #51106115.8.0000.5190 and PlatBr #1449.432. The individual in this manuscript provided an informed consent to publish these case details.

**Key learning points**

- Zika and chikungunya coinfection leads to severe central and peripheral neurological impairment.
- Coinfection with Zika and chikungunya viruses is not related to disease aggravation or patient outcome.
- Health authorities should be made aware of the potential risks of coinfection in endemic areas.

## References

[1] Morrison TE. Reemergence of chikungunya virus. J Virol. 2014; 88:11644–11647. doi: 10.1128/JVI.01432-14 25078691 10.1128/JVI.01432-14 PMC4178719

[2] Roth A, Mercier A, Lepers C, Hoy D, Duituturaga S, et al Concurrent outbreaks of dengue, chikungunya and Zika virus infections—an unprecedented epidemic wave of mosquito-borne viruses in the Pacific 2012–2014. Euro Surveill 2014;19. 10.2807/1560-7917.es2014.19.41.20929 25345518

[3] Brooks JB, Ruiz CA, Fragoso YD. Acute illness with neurological findings caused by coinfection of dengue and chikungunya viruses in a Brazilian patient. J Infect Public Health. 2016. 10.1016/j.jiph.2016.08.010 27616772

[4] Cao-Lormeau VM, Roche C, Teissier A, Robin E, Berry AL, et al Zika virus, French polynesia, South pacific, 2013. Emerg Infect Dis. 2014; 20: 1085–1086. doi: 10.3201/eid2006.140138 24856001 10.3201/eid2006.140138 PMC4036769

[5] Zika virus outbreaks in the Americas. Wkly Epidemiol Rec. 2015;90: 609–610. 26552108

[6] Villamil-Gomez WE, Rodriguez-Morales AJ, Uribe-Garcia AM, Gonzalez-Arismendy E, Castellanos JE, et al Zika, dengue, and chikungunya co-infection in a pregnant woman from Colombia. Int J Infect Dis. 2016. 10.1016/j.ijid.2016.07.017 27497951

[7] Zambrano H, Waggoner JJ, Almeida C, Rivera L, Benjamin JQ, et al Zika Virus and Chikungunya Virus CoInfections: A Series of Three Cases from a Single Center in Ecuador. Am J Trop Med Hyg. 2016. 10.4269/ajtmh.16-0323 PMC5062796 27402518

[8] Weaver SC, Costa F, Garcia-Blanco MA, Ko AI, Ribeiro GS, et al Zika virus: History, emergence, biology, and prospects for control. Antiviral Res. 2016;130: 69–80. doi: 10.1016/j.antiviral.2016.03.010 26996139 10.1016/j.antiviral.2016.03.010 PMC4851879

[9] Duffy MR, Chen TH, Hancock WT, Powers AM, Kool JL, et al Zika virus outbreak on Yap Island, Federated States of Micronesia. N Engl J Med. 2009;360: 2536–2543. doi: 10.1056/NEJMoa0805715 19516034 10.1056/NEJMoa0805715

[10] Brito C. Zika Virus: A New Chapter in the History of Medicine. Acta Med Port. 2015;28: 679–680. 26849748 10.20344/amp.7341

[11] Faria NR, Azevedo Rdo S, Kraemer MU, Souza R, Cunha MS, et al Zika virus in the Americas: Early epidemiological and genetic findings. Science. 2016;352: 345–349. doi: 10.1126/science.aaf5036 27013429 10.1126/science.aaf5036 PMC4918795

[12] Franca RF, Neves MH, Ayres CF, Melo-Neto OP, Filho SP. First International Workshop on Zika Virus Held by Oswaldo Cruz Foundation FIOCRUZ in Northeast Brazil March 2016—A Meeting Report. PLoS Negl Trop Dis. 2016;10: e0004760 doi: 10.1371/journal.pntd.0004760 27258065 10.1371/journal.pntd.0004760 PMC4892518

[13] Zinszer K, Morrison K, Brownstein JS, Marinho F, Santos AF, et al Reconstruction of Zika Virus Introduction in Brazil. Emerg Infect Dis. 2017;23: 91–94. doi: 10.3201/eid2301.161274 27618573 10.3201/eid2301.161274 PMC5176213

[14] Pessoa R, Patriota JV, Lourdes de Souza M, Felix AC, Mamede N, et al Investigation Into an Outbreak of Dengue-like Illness in Pernambuco, Brazil, Revealed a Cocirculation of Zika, Chikungunya, and Dengue Virus Type 1. Medicine (Baltimore). 2016;95: e3201. 27015222 10.1097/MD.0000000000003201 PMC4998417

[15] Chandak NH, Kashyap RS, Kabra D, Karandikar P, Saha SS, et al Neurological complications of Chikungunya virus infection. Neurol India. 2009;57:177–180. doi: 10.4103/0028-3886.51289 19439849 10.4103/0028-3886.51289

[16] Cao-Lormeau VM, Blake A, Mons S, Lastere S, Roche C, et al Guillain-Barre Syndrome outbreak associated with Zika virus infection in French Polynesia: a case-control study. Lancet. 2016;387: 1531–1539. doi: 10.1016/S0140-6736(16)00562-6 26948433 10.1016/S0140-6736(16)00562-6 PMC5444521

[17] Chusri S, Siripaitoon P, Hirunpat S, Silpapojakul K. Case reports of neuro-Chikungunya in southern Thailand. Am J Trop Med Hyg. 2011;85: 386–389. doi: 10.4269/ajtmh.2011.10-0725 21813863 10.4269/ajtmh.2011.10-0725 PMC3144841

[18] Mercado M, Acosta-Reyes J, Parra E, Pardo L, Rico A, et al Clinical and histopathological features of fatal cases with dengue and chikungunya virus co-infection in Colombia, 2014 to 2015. Euro Surveill. 2016;21. 10.2807/1560-7917.ES.2016.21.22.30244 27277216

[19] Waggoner JJ, Gresh L, Vargas MJ, Ballesteros G, Tellez Y, et al Viremia and Clinical Presentation in Nicaraguan Patients Infected with Zika Virus, Chikungunya Virus, and Dengue Virus. Clin Infect Dis. 2016. 10.1093/cid/ciw589 PMC5146717 27578819

[20] Mathias CJ, da Costa DF, Fosbraey P, Bannister R, Wood SM, et al Cardiovascular, biochemical and hormonal changes during food-induced hypotension in chronic autonomic failure. J Neurol Sci. 1989;94: 255–269. 2693619 10.1016/0022-510x(89)90235-9

[21] Taraphdar D, Sarkar A, Mukhopadhyay BB, Chatterjee S. A comparative study of clinical features between monotypic and dual infection cases with Chikungunya virus and dengue virus in West Bengal, India. Am J Trop Med Hyg. 2012;86: 720–723. doi: 10.4269/ajtmh.2012.11-0704 22492160 10.4269/ajtmh.2012.11-0704 PMC3403770

[22] Omarjee R, Prat C, Flusin O, Boucau S, Tenebray B, et al Importance of case definition to monitor ongoing outbreak of chikungunya virus on a background of actively circulating dengue virus, St Martin, December 2013 to January 2014. Euro Surveill. 2014;19. 10.2807/1560-7917.es2014.19.13.20753 24721537

[23] Renault P, Solet JL, Sissoko D, Balleydier E, Larrieu S, et al A major epidemic of chikungunya virus infection on Reunion Island, France, 2005–2006. Am J Trop Med Hyg. 2007;77: 727–731. 17978079

[24] Tandale BV, Sathe PS, Arankalle VA, Wadia RS, Kulkarni R, et al Systemic involvements and fatalities during Chikungunya epidemic in India, 2006. J Clin Virol. 2009;46: 145–149. doi: 10.1016/j.jcv.2009.06.027 19640780 10.1016/j.jcv.2009.06.027

